# Attitudes and Intention to Work With Older Adults by USA and Israeli Nursing Students: Measure Adaptation and Testing

**DOI:** 10.1093/geroni/igaa057.700

**Published:** 2020-12-16

**Authors:** Catherine Van Son, Anna Zisberg, Ksenya Shulyaev

**Affiliations:** 1 Washington State University, Vancouver, Washington, United States; 2 Faculty of Social Welfare and Health Sciences, University of Haifa, Haifa, Israel, Haifa, Isreal, United States; 3 University of Haifa, Mount Carmel, Hefa, Israel

## Abstract

More nurses are needed to meet the healthcare needs of older adults. Valid and reliable measures are lacking that assess attitudes and intentions of nursing students to select a career in geriatrics. This paper discusses the applicability of the Carolina Opinions on Care of Older Adults (COCOA) instrument on attitudes towards and intention to work with older adults among nursing students. A cross-sectional survey design was used with samples of nursing students from the United States (N=122) and Israel (N=109). Students completed the COCOA instrument and the measure’s dimensionality, construct validity, reliability and equivalence among the nursing students was tested. Exploratory Factor Analysis (EFA) produced 5 factors explaining 59% of the variation with most of the items (17, 71%) loading above 0.40 on subscales according to the instrument’s original structure. Four items loaded on different subscales than originally described. One original subscale was eliminated (Value of Older Adults), and one new subscale was created (Older versus Younger Adults). The reliability scores were good to acceptable for all subscales (.60 - .80). Confirmatory FA supported the data’s fit to the modified COCOA instrument (CMIN/DF=1.55, CFI=.93, IFI=.93, RMSEA=.05). The analysis of model equivalence for USA and Israeli samples revealed significant differences attributed to Experience in Caring for Older Adults subscale. The modified COCOA instrument demonstrated good construct validity and reliability and can serve as estimation of nursing students’ attitudes towards older adults and intention to choose geriatrics as a career. Further studies are needed to test its predictive validity.

